# Mitochondrial DNA Variation Correlated With the High Altitude Intolerance in Chinese Young Han Males

**DOI:** 10.3389/fcvm.2022.832136

**Published:** 2022-02-25

**Authors:** Zongbin Li, Chunwei Liu, Jun Guo, Yajun Shi, Yang Li, Jinli Wang, Shanshan Zhou, Yundai Chen

**Affiliations:** ^1^Department of Cardiology, The Sixth Medical Center of Chinese PLA General Hospital, Beijing, China; ^2^The Second School of Clinical Medicine, Southern Medical University, Guangzhou, China; ^3^Department of Cardiology, The First Medical Center of Chinese PLA General Hospital, Beijing, China

**Keywords:** acute mountain sickness, hypoxia, mutation, mitochondrial dysfunction, altitude intolerance

## Abstract

**Objective:**

Acute exposure to hypobaric hypoxia can trigger acute mountain sickness (AMS), while the exact mechanism has not been fully revealed. The role of genetic factors in the susceptibility of various high-altitude diseases has also gained much interest. Previous studies have provided evidence for the link between AMS and certain nuclear genes or mitochondrial haplogroup. The correlation between point mutations of mitochondrial DNA (mtDNA) and AMS was further explored in the present study.

**Methods:**

A total of 84 young Han males residing at low altitude were taken to an elevation of 4,000 m within 40 h. We collected data of their heart rate, blood pressure, peripheral oxygen saturation (SaO_2_), and obtained blood samples, at sea level and at high altitude. AMS was diagnosed using the revised version of the Lake Louise Questionnaire Score. Sequencing was utilized to identify the association between mtDNA alleles and the occurrence of AMS. We also assessed the association between the presence of AMS and physiological variables, and provided a preliminary discussion of the association between genotypic and phenotypic variation.

**Results:**

The percentage of neutrophils [Odds ratio (OR): 1.06, 95% confidence interval (CI): 1.01–1.12, *P* = 0.034) and SaO_2_ level (OR: 0.87, 95% CI: 0.79–0.95, *P* = 0.004) were independently associated with the development of AMS. A4576G was a risk factor for AMS (OR: 6.27, 95% CI: 1.2–32.7). T11613C (OR: 0.10, 95% CI: 0.01–0.83), A8923G (OR: 0.15, 95% CI: 0.03–0.76), and T5543C (OR: 0.19, 95% CI: 0.04–0.95) were protective factors for AMS. The level of SaO_2_ was significantly lower in the individual with A4576G mutation as compared with the individual without A4576G mutation (68.1 ± 7.9 vs. 75.8 ± 6.1, *P* = 0.001). The level of serum sodium was significantly higher in the individual with A8923G mutation as compared to the individual without A8923G mutation (144.6 ± 1.9 vs. 143.2 ± 1.9, *P* = 0.027).

**Conclusions:**

The increase in neutrophils and the disability to preserve oxygen saturation may be associated with the high altitude intolerance in young Chinese Han males. A4576G is the risk factor for AMS. T11613C, A8923G, and T5543C are protective factors for AMS. The role of A8923G mutation may correlate with the sodium and water balance and the role of the A4576G mutation may be related to the disability to maintain blood oxygen level after quickly entering the plateau.

## Introduction

Following the expansion of railway and air travel in Qinghai and Tibet, increasing numbers of people travel to high altitudes for various reasons, such as mining, tourism, trekking, and deployment. For people living in the plains for a long time, one of the most worrying problems is the possible occurrence of acute altitude sickness after entering the plateau. Acute altitude sickness is a general term for acute mountain sickness (AMS), high altitude brain edema, and high altitude pulmonary edema that may occur when the altitude exceeds 2,500 m. Among them, AMS has the highest incidence, which can reach 10% at an altitude of 2,500 m, and increases with the elevation (1). The main manifestations of AMS are headache, dizziness, fatigue, and symptoms in the digestive system.

The specific pathogenesis of AMS has not been fully elucidated. Since 2010, a breakthrough has been made in the gene research of Tibetan people's adaptation to high altitude (2). The research on the genetic mechanism has been carried out more widely. At present, the research on plateau genetics is mainly focused on two aspects: one is the study of plateau adaptability gene of people living in plateaus for generations, and the other is the study of plateau disease susceptibility gene of people living in the plains. Several key genes related to high altitude adaptability also showed a certain correlation in the study of susceptibility to acute high altitude disease. Recently, the mitochondrial genome has also attracted some attention due to its genetic characteristics. Mitochondrial nt3010G-nt3970C haplotype and haplogroup M9a1a1c1b are implicated in high-altitude adaptation of Tibetans (3, 4). Mitochondrial DNA (mtDNA) haplogroups D and M9 are related to individual tolerance to AMS, while haplogroups M7 and B are risk factors for AMS (5, 6). However, there are a few reports about the relationship between AMS and mtDNA point mutations.

In the present study, we sequenced the mitochondrial genome and identified the mutation sites in the blood samples of young male volunteers of Han nationality, who rushed to the plateau, and detected some physiological indexes in the plains and plateau environment. The relationship between AMS and mtDNA point mutation was analyzed. We also explored the physiological characteristics related to AMS through the comparison of physiological indexes and sought the possible target of mitochondrial gene mutation.

## Materials and Methods

### Participants

This project included 84 Chinese male adults aged 18 to 35 years, none of whom had any experience of exposure to high altitude. Other exclusion criteria included any history of primary headache, cerebral neoplasm, heart failure, or chronic obstructive pulmonary disease. This study was approved by the Institutional Review Board (IRB) of the Chinese PLA General Hospital. The IRB-approved number is “S2014-070-01”. The individuals in this manuscript given written informed consent and a verbal explanation concerning the study prior to obtaining the written informed consent for their participation.

### Study Design

Participants' demographic data were first collected, including age, height, weight, and body mass index (BMI). Physiological measurements performed at altitude were also performed, prior to departure, at sea level (Beijing). After collecting these baseline data, the participants were taken to Geermu, Qinghai Province (2,800 m), by train for 30 h. After several hours of waiting at Geermu, these participants were taken to the destination (4,000 m) by bus within 3 h. The recording of dynamic electrocardiogram and ambulatory blood pressure monitoring were started at an altitude of 2,800 m and terminated at the destination. About 6–8 h after arrival at the altitude of 4,000 m, Lake Louise Questionnaire was completed. Over the next 2 h, peripheral oxygen saturation (SaO_2_) was measured and blood draw was performed. Blood samples were analyzed for hemoglobin concentration, white blood cells and their subsets, platelet, liver and kidney function, serum electrolyte, and myocardial damage marker. The classic version of the Lake Louise Questionnaire includes such symptoms as headache, gastrointestinal symptoms (anorexia, nausea, or vomiting), fatigue/weakness, and dizziness/lightheadedness. The threshold was set as three points, and headache was a necessary symptom (7).

### Mutational Analysis

Genomic DNA was isolated from the whole blood of participants using Genomic DNA Isolation Kits (Thermo Fisher Scientific, Minneapolis Massachusetts, MA). The mitochondrial genome was enriched and purified by a long polymerase chain reaction (PCR) using human mitochondrial genome-specific primers. The primer sequences are as follows: forward 1, 5′-GACGGGCTCACATCACCCCATAA-3′, reverse 1, 5′-GCGTACGGCCAGG GCTATTGGT-3′; Forward 2, 5′-GGTGGCTGGCACGAAATTGACC-3′, reverse 2, 5′-GCCACAACTAACCTCCTCGGACTCCT-3′. The amplified products were segmented by ultrasonic mechanical method to 100–500 bp. Sequencing Libraries were prepared based on the protocols of SureSelect QXT Library Prep Kit (Agilent, California, CA). Exome capture was prepared based on the protocols of Agilent SureSelect QXT Target Enrichment for Illumina Multiplexed Sequencing version E0. Before hybridization, quantity and quality of library DNA were assessed by Agilent 2200 TapeStation. The DNA libraries were mixed with capture probes of targeted regions using the SureSelect Human All Exon V6 kit. The hybridization was performed at 65°C for 1 h to ensure that the targeted regions bound to the capture probes thoroughly. Streptavidin beads were used to capture probes containing the targeted regions of interest. The enriched libraries on the beads were then amplified by PCR. The enriched libraries were cleaned with Ampure XP beads (Agencourt, Boston, MA, USA) according to the SureSelect QXT protocol, and then validated by Agilent 2200 TapeStation and qPCR for quality control analysis. The libraries were denatured and diluted to optimal concentration and applied in the cluster generation steps. Illumina NovaSeq 6000 S2 Reagent Kit (300 cycles) was used for paired-end 2 × 150 bp sequencing on an Illumina NovaSeq 6000 System.

The genomic blast comparison of the designed primers showed that the amplified product sequence of the primers was indeed on the mitochondrial genome. The present sequencing method directly captures mtDNA in the human genome, which greatly reduces the impact of nuclear DNA on mtDNA detection. The sequence obtained by sample sequencing was compared with the revised Cambridge reference sequence (rCRS, GenBank NC_012920.1) in the database. GATK analysis software was used to count the reading and total data yield of sequencing fragments, and evaluate the sequencing depth, coverage, and uniformity. Each cycle of all sequencing reads was monitored to check the sample and sequencing quality. The sequencing quality analysis showed that 99.14% of reads sequencing quality values were greater than Q30.

### Statistical Analyses

Data were expressed as means ± SD unless otherwise stated. Differences between the two groups were analyzed with Student's *t*-test. The qualitative data was expressed by the number of cases (percentage), and the quantitative data of non-normal distribution was expressed by median (p 25, p 75). The Chi-square test or exact probability method was used to compare qualitative data. A *P* < 0.05 was considered statistically significant. Statistical analysis was performed with SPSS Statistics (IBM, USA).

## Results

### Demographic Analysis

According to the revised Lake Louise criteria, 34 out of 84 volunteers met the AMS diagnostic criteria, indicating that 40.5% of the volunteers in this study developed AMS. The average age of the volunteers with AMS was significantly lower than that without AMS. There was no significant difference in height, weight, and BMI between the two groups of volunteers (Table 1).

**Table 1:** Demographic data of the subjects in two groups.

**Variables**	**AMS+ (*N* = 34)**	**AMS– (*N* = 50)**	***P*-value**
Age (years)	25 (24, 26)	26 (24, 27)	0.036^*^
Weight (kg)	71.6 ± 9.0	71.4 ± 8.4	0.501
Height (cm)	175.3 ± 4.7	174.6 ± 4.7	0.926
BMI (kg/m^2^)	23.0 (21.6, 24.5)	23.4 (21.3, 25.1)	0.575

*BMI represents body mass index*.

* *Represent P < 0.05*.

### Ambulatory Electrocardiogram

There was no significant difference between the individuals with AMS and without AMS in the comparison of the highest heart rate, lowest heart rate, average heart rate, and heart rate variability (HRV) index (Table 2).

**Table 2:** Comparison of ambulatory electrocardiogram at high altitude between two groups of volunteers.

**Variables**	**AMS+ (*n* = 34)**	**AMS– (*n* = 50)**	***P-*value**
HR_Max_ (bpm)	134 ± 12	133 ± 16	0.878
HR_Min_ (bpm)	53 ± 7	52 ± 7	0.382
HR_mean_ (bpm)	82 ± 8	81 ± 10	0.590
HRV index	35.3 ± 10.7	39.4 ± 10.6	0.125

*HR_Max_ represents maximum heart rate; HR_Min_ represents minimum heart rate; HR_mean_ represents mean heart rate; HRV represents heart rate variability*.

### Ambulatory Blood Pressure Examination

There was no significant difference in the comparison of mean systolic blood pressure, mean diastolic blood pressure, blood pressure coefficient of variation, and blood pressure load between the individuals with AMS and without AMS (Table 3).

**Table 3:** Comparison of ambulatory blood pressure examination at high altitude between two groups of volunteers.

**Variables**	**AMS+ (*n* = 34)**	**AMS– (*n* = 50)**	***P*-value**
Mean SBP(mmHg)	119.6 ± 8.3	119.5 ± 8.1	0.983
Mean DBP(mmHg)	75.4 ± 7.1	74.4 ± 6.8	0.526
Coefficient of variation of 24 h SBP	15.6 ± 5.3	16.6 ± 6.0	0.424
Coefficient of variation of 24 h SBP	25.0 (16.6, 29.8)	24.1 (17.7, 31.6)	0.936
24 h SBP load (%)	21.9 (16.7, 29.7)	18.1 (11.6, 31.4)	0.241
24 h DBP load (%)	33.3 (23.7, 42.8)	30.3 (18.4, 38.1)	0.226

*SBP represents systolic blood pressure; DBP represents diastolic blood pressure*.

### Blood Routine Examination

The percentage of neutrophils in individuals with AMS was significantly higher than that of those without AMS (64.5 ± 11.2% vs. 58.1 ± 8.8%, *P* = 0.014), while the percentage of monocytes and lymphocytes was lower than that of those without AMS. There was no statistical difference between the two groups in the comparison of the above indicators in the plains environment. There was no significant difference in hemoglobin and platelet count between the two groups (Table 4).

**Table 4:** Comparison of blood routine test results at high altitude between two groups of volunteers.

**Variables**	**AMS+ (*n* = 34)**	**AMS– (*n* = 50)**	***P*-value**
RBC (10^9^/L)	5.3 ± 0.3	5.2 ± 0.4	0.050
Hb (g/L)	164.2 ± 7.5	161.0 ± 8.6	0.075
MCH (pg)	30.7 ± 1.3	31.0 ± 1.0	0.270
MCHC (g/L)	343.0 (334.0, 346.0)	339.5 (333.0, 342.0)	0.124
MCV (fl)	91.1 (88.8, 92.9)	91.9 (89.3, 93.1)	0.202
WBC (10^9^/L)	8.7 ± 2.5	8.1 ± 1.7	0.248
Neutrophil (%)	64.5 ± 11.2	58.1 ± 8.8	0.014^*^
Monocyte (%)	4.0 (3.7, 4.5)	4.7 (4.0, 6.0)	<0.001^*^
Basophil (%)	0.5 (0.4, 0.8)	0.6 (0.3, 0.7)	0.929
Lymphocyte (%)	29.3 ± 10.5	34.9 ± 8.1	0.008^*^
Eosinophil (%)	1.2 (0.8,2.1)	1.3 (0.7,1.9)	0.805
Platelet (10^9^/L)	220.6 ± 52.8	232.4 ± 45.4	0.283

*RBC represents red blood cell; Hb represents hemoglobin; MCH represents mean corpuscular hemoglobin; MCHC represents mean corpuscular hemoglobin concentration; MCV represents mean corpuscular volume; Plt represents platelet; WBC represents white blood cell*.

* *Represents statistically significant comparation between AMS+ group and AMS– group*.

### Blood Biochemical Test

There was no statistical difference between the two groups in the comparison of biochemical indexes, such as liver and kidney function, lipid metabolism, electrolyte, and myocardial injury markers (Table 5).

**Table 5:** Comparison of blood biochemistry at high altitude between two groups of volunteers.

**Variables**	**AMS+ (*n* = 34)**	**AMS– (*n* = 50)**	***P-*value**
ALT (U/L)	16.0 (13.8, 26.5)	17.5 (13.8, 25.3)	0.784
AST (U/L)	18.0 (14.0, 21.3)	16.0 (15.0, 19.0)	0.546
AKP (U/L)	70.0 (61.8, 77.0)	75.0 (61.8, 89.5)	0.327
Total protein (g/L)	78.3 ± 3.9	77.5 ± 4.2	0.404
Albumin (g/L)	51.6 ± 2.2	52.1 ± 2.5	0.339
Urea nitrogen (mmol/L)	5.1 (4.6, 5.5)	4.8 (4.3, 5.4)	0.559
Creatinine (μmol/L)	84.3 ± 12.8	79.4 ± 11.5	0.071
Cholesterol (mmol/L)	4.3 ± 0.7	4.5 ± 0.6	0.119
HDL(mmol/L)	1.3 ± 0.3	1.4 ± 0.3	0.531
LDL (mmol/L)	2.5 ± 0.7	2.7 ± 0.6	0.364
T-Bil (μmol/L)	6.6 (5.5, 9.9)	7.7 (5.7, 11.0)	0.360
D-Bil (μmol/L)	2.7 (2.1, 3.4)	3.1 (2.5, 3.9)	0.106
TNT (μg/L)	0.004 (0.004, 0.005)	0.004 (0.003, 0.006)	0.825
Sodium (mmol/L)	143.0 (142.0, 144.0)	144.0 (142.0, 145.0)	0.138
Chloride (mmol/L)	101.6 (100.7, 104.0)	102.8 (101.8, 103.7)	0.179

*ALT represents alanine aminotransferase; AST represents aspartate aminotransferase; AKP represents alkaline phosphatase; HDL represents high density lipoprotein; LDL represents low density lipoprotein; Plt represents platelet; T-BIL represents total bilirubin. D-BIL represents Direct bilirubin; TNT represents troponin T*.

### Logistic Regression Analysis of Clinical Indicators

The variables with *P* < 0.10 in univariate analysis were further included in logistic regression analysis. In binary logistic regression analysis, neutrophil percentage (OR: 1.06, 95% CI: 1.01–1.12, *P* = 0.034) and pulse oxygen saturation (OR: 0.87, 95% CI: 0.79–0.95, *P* = 0.004) still reflected the correlation with AMS (Table 6).

**Table 6:** Logistic regression analysis of demographic data and biological indicators.

**Variables**	**OR**	**95% CI**	**P-value**
Age (years)	0.82	0.63–1.08	0.157
BMI (kg/m^2^)	0.91	0.74–1.12	0.376
Neutrophil (%)	1.06	1.01–1.12	0.034^*^
SaO_2_ (%)	0.87	0.79–0.95	0.004^*^
Hb (g/L)	1.05	0.98–1.12	0.200
Creatinine (mmol/L)	1.02	0.97–1.06	0.459

*BMI represents body mass index; SaO2 represents oxygen saturation; Hb represents hemoglobin*.

* *Represents P < 0.05*.

### Mitochondrial Gene Mutation Analysis

There was a statistical difference between the two groups in the comparison of the number of mitochondrially encoded NADH dehydrogenase 3 (mt-ND3) gene mutations (Table 7). In the comparison of single mutation site, two groups of volunteers had statistical differences in the comparison of 21 mutation sites, including three gene sites encoding tRNA, four gene sites encoding rRNA, and 14 gene sites encoding subunits. Two loci were only found in the individuals with AMS, eight loci were only found in the individuals without AMS, and a total of 11 loci were found in both groups (Table 8).

**Table 7:** Comparison of mitochondrial gene mutation rates between the two groups.

**Gene locus**	**AMS+ (*n* = 34)**	**AMS– (*n* = 50)**	***P-*value**
MT-ND1	34 (100%)	49 (98%)	1.000
MT-ND2	34 (100%)	47 (94%)	0.392
MT-ND3	34 (100%)	41 (82%)	0.024^*^
MT-ND4	30 (88%)	41 (82%)	0.438
MT-ND4L	14 (41%)	23 (46%)	0.662
MT-ND5	33 (97%)	48 (96%)	1.000
MT-ND6	29 (85%)	40 (80%)	0.534
MT-CYB	34 (100%)	49 (98%)	1.000
MT-CO1	33 (97%)	48 (96%)	1.000
MT-CO2	31 (91%)	44 (88%)	0.918
MT-CO3	31 (91%)	44 (88%)	0.918
MT-ATP6	34 (100%)	50 (100%)	-
MT-ATP8	34 (100%)	44 (88%)	0.096
MT-RNR1	34 (100%)	50 (100%)	-
MT-RNR2	34 (100%)	50 (100%)	-
MT-TA	21 (62%)	32 (64%)	0.835
MT-TC	24 (71%)	32 (64%)	0.530
MT-TD	19 (56%)	31 (62%)	0.575
MT-TE	12 (35%)	25 (50%)	0.183
MT-TF	32 (94%)	48 (96%)	1.000
MT-TG	14 (41%)	29 (58%)	0.130
MT-TH	17 (50%)	31 (62%)	0.275
MT-TI	24 (71%)	31 (62%)	0.416
MT-TK	20 (59%)	29 (58%)	0.940
MT-TM	12 (35%)	20 (40%)	0.663
MT-TN	16 (47%)	26 (52%)	0.657
MT-TP	24 (71%)	34 (68%)	0.801
MT-TQ	24 (71%)	36 (72%)	0.888
MT-TR	11 (32%)	22 (44%)	0.283
MT-TS1	21 (62%)	24 (48%)	0.214
MT-TS2	16 (47%)	23 (46%)	0.924
MT-TT	25 (74%)	40 (80%)	0.487
MT-TV	20 (59%)	29 (58%)	0.940
MT-TW	26 (76%)	30 (60%)	0.116
MT-TY	11 (32%)	21 (42%)	0.371
MT-TL1	22 (65%)	35 (70%)	0.610
MT-TL2	18 (53%)	27 (54%)	0.924

* *Represents P < 0.05*.

**Table 8:** Differences in mitochondrial gene mutation sites between the two groups.

**Mutation sites**	**AMS+ (*n* = 34)**	**AMS– (*n* = 50)**	***P-*value**
**tRNA**
T15968C	3 (8.8%)	14 (28%)	0.032
T5543C	2 (5.9%)	11 (22%)	0.045
A12320G		9 (18%)	0.024
**rRNA**
A1713G	2 (5.9%)	11 (22%)	0.045
A2135G	6 (17.6%)		0.008
A2270G		8 (16%)	0.038
A1864G		8 (16%)	0.038
**Subunit**
T11613C	1 (2.9%)	12 (24%)	0.009
T14063C	1 (2.9%)	11 (22%)	0.033
T14933C	3 (8.8%)	13 (26%)	0.049
A4576G	7 (20.6%)	2 (4%)	0.040
A6891G	1 (2.9%)	11 (22%)	0.030
T7751C	1 (2.9%)	12 (24%)	0.009
A8923G	2 (5.8%)	13 (26%)	0.018
A9681G	5 (14.7%)	1 (2%)	0.038
G9055A	4 (11.8%)		0.024
T14060C		8 (16%)	0.038
T14502C		8 (16%)	0.038
T15404C		10 (20%)	0.015
G5910A		8 (16%)	0.038
T6481C		8 (16%)	0.038

### Conservation Analysis

The conservation index of 13 sites among the 21 mutation sites met the standard of 0.80, including 2 gene sites encoding tRNA, 2 gene sites encoding rRNA, and 9 gene sites encoding subunits. After adjusting with age and BMI, there were still statistical differences in four loci (A4576G, T11613C, A8923G, and T5543C). A4576G mutation lies in mitochondrially encoded NADH dehydrogenase 2 (mt-ND2) gene, T11613C mutation lies in mitochondrially encoded NADH dehydrogenase 4 (mt-ND4) gene, A8923G lies in mt-ATPase6 gene, and T5543C mutation site lies in the gene locus encoding tRNA. Among the four loci, A4576G was considered as the risk factor of AMS (OR: 6.27, 95% CI: 1.2–32.7), and A8923G (OR: 0.15, 95% CI: 0.03–0.76), T11613C (OR: 0.10, 95% CI: 0.01–0.83), and T5543C (OR: 0.19, 95% CI: 0.0–0.95) as the protective factors of AMS (Tables 9, 10).

**Table 9:** Mutation sites with conservation index ≥ 0.80.

**Mutation site**	**AMS+ (*n* = 34)**	**AMS– (*n* = 50)**	**Gene locus**	**Conservative index**
T5543C	2 (5.9%)	11 (22%)	MT-TW	1
A12320G	0	9 (18%)	MT-TL2	1
A2135G	6 (17.6%)	0	MT-RNR2	0.98
A2270G	0	8 (16%)	MT-RNR2	0.98
A4576G	7 (20.6%)	2 (4%)	MT-ND2	0.96
T11613C	1 (2.9%)	12 (24%)	MT-ND4	1
T14502C	0	8 (16%)	MT-ND6	0.81
T14933C	3 (8.8%)	13 (26%)	MT-CYB	1
T15404C	0	10 (20%)	MT-CYB	1
T6481C	0	8 (16%)	MT-CO1	1
A9681G	5 (14.7%)	1 (2%)	MT-CO3	0.94
A8923G	2 (5.8%)	13 (26%)	MT-ATP6	1
G9055A	4 (11.8%)	0	MT-ATP6	0.87

**Table 10:** Logistic regression analysis of mitochondrial gene mutation.

**Mutation site**	**Gene locus**	**AMS+ (*n* = 34)**	**AMS– (*n* = 50)**	**OR**	**95%CI**	***P-*value**
T5543C	MT-TW	2 (5.9%)	11 (22%)	0.19	0.04–0.95	0.044
A4576G	MT-ND2	7 (20.6%)	2 (4%)	6.27	1.2–32.7	0.029
T11613C	MT-ND4	1 (2.9%)	12 (24%)	0.10	0.01–0.83	0.033
A8923G	MT-ATP6	2 (5.8%)	13 (26%)	0.15	0.03–0.76	0.022

### Pulse Oxygen Saturation

**Figure 1:**
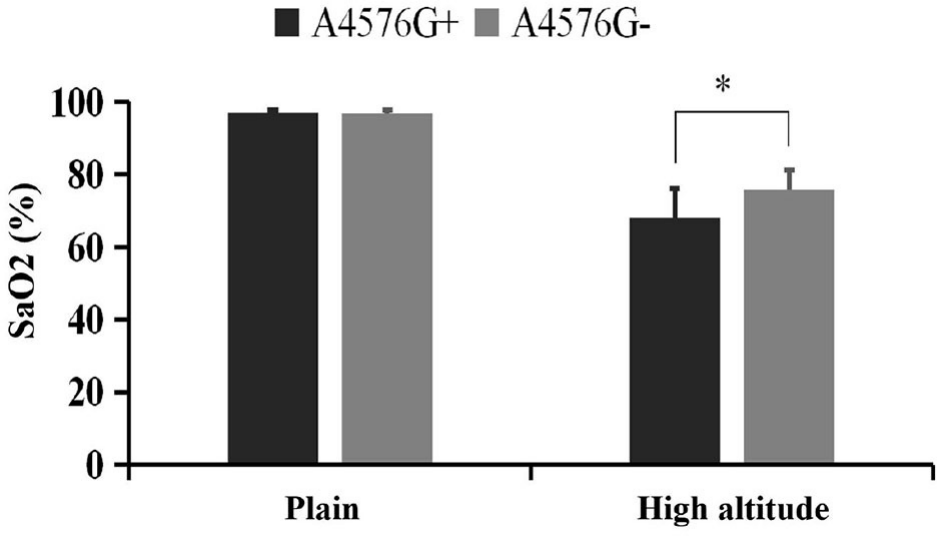
Comparison of pulse oxygen saturation between A4576G+ and A4576G- groups. *Represents statistically significant comparation between A4576G+ group and A4576G- group at high altitude.

The level of pulse oxygen saturation in the volunteers with A4576G mutation was significantly lower than that in the volunteers without A4576G mutation (68.1 ± 7.9% vs. 75.8 ± 6.1%, *P* = 0.001), and there was no significant difference between the two groups in the plains environment (Figure 1).

### Serum Sodium

**Figure 2:**
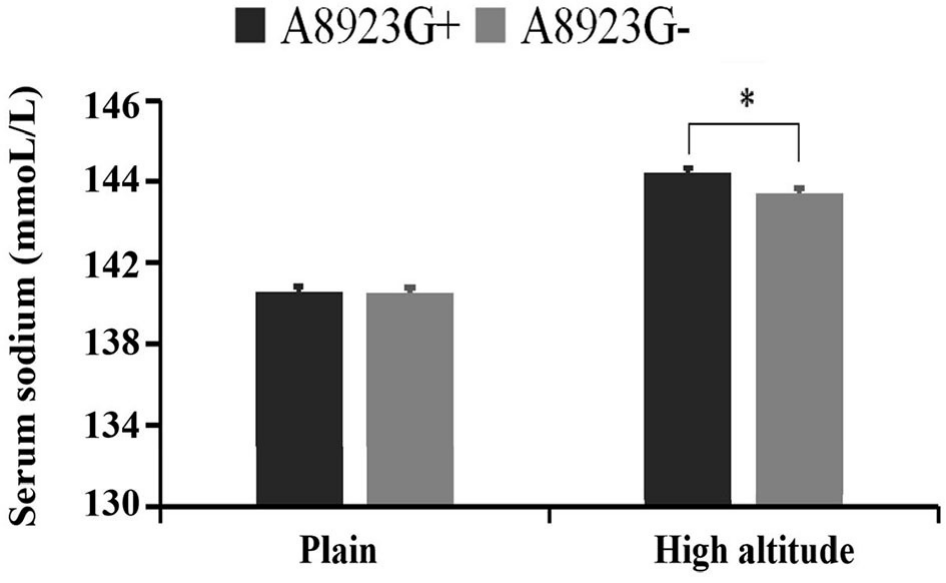
Comparison of blood sodium concentration between A8923G+ and A8923G- groups. *Represents statistically significant comparation between A8923G+ group and A8923G- group at high altitude.

The serum sodium level of the volunteers with A8923G mutation was significantly higher than that of the volunteers without the mutation (144.6 ± 1.9 mmol/L vs. 143.2 ± 1.9 mmol/L, *P* = 0.027), and there was no significant difference between the two groups in the plains environment (Figure 2).

## Discussion

Acute mountain sickness is a hitherto unsolved problem, affecting the individuals who long inhabited the plains when they quickly reached the plateau (8, 9). Previous studies have estimated that over 10% of travelers experienced various degrees of AMS when ascending an elevation of ≥2,500 m (10–12). The incidence of acute altitude reaction increased with increase in altitude. At ~4,000 m, the incidence of AMS has been estimated at 36.7–47.5% (12, 13). The rate of AMS at 40.5% in the current study is generally consistent with the previous reports. The diversity of different research results among the studies may reflect differences in a variety of factors, including gender and age of the enrolled individuals, the speed of ascent, arrival altitude, timing of AMS assessment, as well as the field conditions.

In the present study, approximately 40.5% of the individuals developed AMS. The main reason for altitude reaction is hypoxia. The development of AMS is directly related to human tolerance to hypoxia. Compared with thin and short people, strong or fat people have higher oxygen consumption. Under the same environment, the higher the degree of hypoxia, the stronger is the altitude reaction. Similarly, the younger the age, the more the metabolism and energy consumption, the greater the body's oxygen consumption, and the stronger the altitude reaction. The results of this study suggest that the volunteers with AMS are younger than those without AMS. The data leading to the notion that age may be protective against AMS was previously reported (14–16).

In the present study, the percentage of neutrophils in the subjects with AMS was higher than that of the volunteers without AMS, while both were still in the normal range. Neutrophils are an important part of the innate immunity of the human body, forming the first line of defense for the body to resist infection, tissue damage, and other processes (17). Neutrophils can be activated by cytokines, free radicals, and other activating factors in the complex inflammatory response network, and further release inflammatory mediators and oxygen free radicals, amplifying the inflammatory response, which undoubtedly plays an important role in the above process. The mechanism for the increase of neutrophils in the AMS population needs to be further explored. The decrease of the percentage of monocytes and lymphocytes in the individuals with AMS may be the result of the relative increase of neutrophils.

Another physiological index related to AMS occurrence in our present study is pulse oxygen saturation level. In the plateau environment, atmospheric pressure decreases with altitude, and the oxygen partial pressure of inhaled gas will also decrease significantly. Studies have shown that low blood oxygen saturation is related to the occurrence of AMS, and the oxygen saturation or partial pressure in individuals with AMS is significantly lower than that in those without AMS (9, 18).

Although not statistically significant, we still found that the HRV index was lower in the individuals with AMS than in those without AMS. HRV reflects sympathetic and parasympathetic cardiac autonomic nervous system regulation, and a reduction of the HRV is a common finding during acute hypoxia. The previous study showed that the individuals with higher autonomic activity and stronger response to stress had a heavier symptom of AMS, which indicated that HRV can predict AMS to a certain extent (19, 20).

The mutation rate of mt-ND3 gene in the individuals without AMS was significantly lower than that in the individuals with AMS. Mt-ND3 is a subunit of respiratory chain complex I and is involved in the key step of oxidative phosphorylation process. According to previous studies, neutral mutations occur at sites with relatively low conservation, while harmful or adaptive mutations occur at sites with relatively conservative evolution (21). To evaluate the conservation of mutation site species, the conservation index of each site was calculated by using the website mitotool, which calculated the conservation index of mutation site in 43 species including humans. The conservation index of 13 out of 21 loci was ≥0.80, which was considered to be relatively conservative in the evolution of species. Four loci were found to have statistical differences, among which A4576G was considered as the risk factor of AMS, and T11613C, A8923G, and T5543C were considered as the protective factors of AMS. A4576G is the mutation site of the mt-ND2 gene. There is no previous report that A4576G mutation related to the development of the disease. After analysis and correction, there were still differences between the two groups, which were risk factors of AMS. In the further verification of physiological function, there was a significant difference between the two groups in the comparison of pulse oxygen saturation in plateau environment. The level of pulse oxygen saturation of the volunteers with A4576G mutation was significantly lower than that of the volunteers without the mutation, after entering the plateau environment, while the analysis of baseline data showed that there was no significant difference between the two groups in the level of pulse oxygen saturation in the plains environment. Previous studies have also shown that the low oxygen saturation level is related to the occurrence of AMS (9, 18). The mechanism of A4576G mutation as a risk factor of AMS may be related to the reduction in the ability to maintain blood oxygen levels in the high altitude environment.

Like A4576G, T11613C is also located in the gene encoding complex I, which encodes the subunit ND4 of complex I. T11613C mutation resulted in the replacement of leucine at the 285th position of ND4 subunit with proline. T11613C mutation has not been reported in the query of MITOMAP. In this study, T11613C mutation was found in two groups of volunteers, and there were still differences between the two groups after analysis and correction, which were protective factors of AMS. However, in the verification of physiological function, the two groups of volunteers did not show differences in the physiological indicators in this study.

A8923G is the mutation site of the mt-ATPase6 gene which encodes the subunit ATPase6 of mitochondrial respiratory chain ATPase (complex V). ATPase6 mutations usually keep the respiratory chain intact but separate the proton flow from ATP production (22). Therefore, gene mutations of ATPase6-related subunits may affect the production of ATP. A8923G mutation has not been reported to be related to the disease in the query of MITOMAP. In this study, A8923G mutation was found in two groups of volunteers, and there were still differences between the two groups after analysis and correction, which belonged to the protective factor of AMS. In the further functional verification, it was found that there was a significant difference in the serum sodium level between the two groups at high altitude. The blood sodium level of the volunteers with A8923G mutation was significantly higher than that of the volunteers without A8923G mutation, but they were still in the normal range. There was no significant difference between the two groups in the level of blood sodium ion in the plains environment. The level of blood sodium ion is the main component of maintaining the osmotic pressure of plasma crystal. The increase in the osmotic pressure of plasma crystal will lead to the transfer of intracellular fluid to extracellular, thus reducing cell edema. However, the increase of intracranial pressure caused by various causes of cellular edema after entering high altitude hypoxia environment is considered to be one of the mechanisms of AMS (23, 24). In addition, it has been reported that the level of blood sodium in the population without AMS at high altitude was significantly higher than that in the population with AMS, and it may be related to the reduction of cellular edema (25). Therefore, the change of water electrolyte metabolism and the increase of blood sodium level caused by the A8923G mutation may be one of the reasons for protective effect.

T5543C mutation is located in the mitochondrial tryptophan tRNA (mt-TW) gene. Although tRNA only accounts for about 10% of the total coding capacity of mitochondrial genome, more than half of the currently found pathogenic mutations of mitochondrial genes are located in the genes encoding tRNA (26). This may be because the mutations in tRNA genes associated with total protein synthesis have a greater impact than the mutations in a single respiratory chain subunit (22). Mutations in tRNA genes can affect the structure and function of tRNA molecules in various ways, such as affecting tRNA synthesis, maturation, stability, aminoacylation, and interaction with other translation components, leading to different pathogenic mechanisms. Patients with tRNA gene mutations may present with complex multisystem diseases or with single diseases, such as isolated myopathy and cardiomyopathy (27). T5543C is located at the 33rd position in the secondary structure of tRNA, specifically in the anti-codon ring, next to the 5' end of the anti-codon. The base at this position may play an important role in the process of ribose phosphate skeleton rotation and the formation of anti-codon ring. The base at this site is very conservative in evolution, so the mutation at this site may have an important impact on the function of tRNA. T5543C mutations have also been reported to be associated with mitochondrial myopathy in previous studies (27). In this study, T5543C mutation was found in both groups of volunteers. After analysis and correction, there were still differences between the two groups, which belonged to the protective factor of AMS.

The limitations of the present study include recruitment of relatively few volunteers due to traffic and accommodation constraints. The volunteers selected in this study are only young men, which is mainly because the people who go to the plateau to perform tasks are mainly young men. Although we have discovered that the mutation of mitochondrial gene A4576G may be related to the reduction of blood oxygen level and A8923G may be related to water and sodium metabolism in rush to high altitude, further validation is needed at the cellular and animal levels in the future research.

The strengths of the present study are that participants' demographic data were relatively homogenous and they started from low altitude and took the same means of transportation to the plateau at the same time. Next, we used the revised Lake Louise score 2018 for the diagnosis of AMS. The revised Lake Louise score is more accurate at identifying AMS cases. Finally, we comprehensively analyzed the physiological indexes and non-invasive cardiac function indexes of volunteers with and without AMS.

In summary, our present study showed the disability of maintaining blood oxygen saturation level and the increase of neutrophil level related to the occurrence of AMS in Chinese young Han male individuals. The mutation of mitochondrial gene A4576G may be the risk factor for AMS, and its target may be related to disability to maintain blood oxygen level after quickly entering the plateau. T11613C, A8923G, and T5543C mutations are protective factors of AMS in young Han men. A8923G may be related to water and sodium metabolism after quickly entering the plateau.

## Data Availability Statement

The datasets presented in this study can be found in online repositories. The names of the repository/repositories and accession number(s) can be found below: https://www.ncbi.nlm.nih.gov/sra/?term=PRJNA799237.

## Ethics Statement

The studies involving human participants were reviewed and approved by the Ethics Committee of Chinese PLA General Hospital, Chinese People's Liberation Army General Hospital. The patients/participants provided their written informed consent to participate in this study.

## Author Contributions

ZL, CL, JG, and JW completed the experiments. ZL, YS, and YL contributed to the conception, drafted the manuscript, YC contributed to critically revised the manuscript, gave final approval, and agreed to be accountable for all aspects of the work ensuing integrity and accuracy. All authors contributed to the article and approved the submitted version.

## Funding

This study was supported by grants from the National Natural Science Foundation of China (81170249 and 30700305) and the Hygiene and Health Development Scientific Research Fostering Plan of Haidian District of Beijing (HP-2021-03-80602).

## Conflict of Interest

The authors declare that the research was conducted in the absence of any commercial or financial relationships that could be construed as a potential conflict of interest.

## Publisher's Note

All claims expressed in this article are solely those of the authors and do not necessarily represent those of their affiliated organizations, or those of the publisher, the editors and the reviewers. Any product that may be evaluated in this article, or claim that may be made by its manufacturer, is not guaranteed or endorsed by the publisher.
